# Emerging role of lncRNA TP53TG1 as a tumor regulator and biomarker in human malignancies

**DOI:** 10.1007/s12672-026-04752-4

**Published:** 2026-03-09

**Authors:** Bing Wang, Tingting Yu, Lei Luo, Wenqian Tang, Hua Zhang

**Affiliations:** 1 Department of Respiratory and Critical Medicine, Jingmen Hospital of Traditional Chinese Medicine, Jingmen, 448000 P.R. China; 2 Department of Health Management Center, Hubei Provincial Hospital of Traditional Chinese Medicine, Wuhan, 430070 P. R. China; 3 Department of Gastroenterology, Hubei Provincial Hospital of Integrated Chinese and Western Medicine, No. 11, Lingjiao Lake Road, Jianghan District, Wuhan City, 430015 Hubei Province P.R. China

**Keywords:** TP53TG1, Long non-coding RNAs, Cancer, Molecular mechanisms, Diagnosis, Prognosis

## Abstract

Long noncoding RNAs (lncRNAs) are a category of RNA molecules that exceed 200 nucleotides in length and do not possess the ability to encode proteins. Recently, there has been a growing interest among scientists regarding their functions and advancements in research. One notable lncRNA is the tumor protein 53 target gene 1 (TP53TG1), which has emerged in the last few years and is located in the chromosomal region 7q21.12 of the human genome. Studies have increasingly pointed out the role of TP53TG1 in the progression of various cancers, where its expression is altered in several tumors, including hepatocellular carcinoma, breast cancer, gastric cancer, non-small cell lung cancer, colorectal cancer, and glioma. Moreover, extensive research has shown that TP53TG1 acts as a competitive endogenous RNA (ceRNA), engaging in signaling pathways, modulating gene expression, and influencing the proliferation, migration, invasion, apoptosis, epithelial-mesenchymal transition (EMT), and drug resistance of cancer cells. Consequently, TP53TG1 is posited as a significant biomarker for tumor prognosis and is anticipated to be a potentially effective target for cancer therapies. This review article examines the expression, biological roles, and molecular mechanisms of TP53TG1 across various malignancies while exploring its clinical implications.

## Introduction

Due to the elusive nature of cancer and its propensity to reappear, around 10 million people lose their lives to this disease worldwide each year [1]. According to the World Health Organization, it is estimated that by 2024, the yearly total of new cancer cases will climb to 28.9 million [2]. With increasing rates of incidence and mortality linked to cancer, it has become a major public health issue, placing considerable pressure on societies and healthcare systems across the globe. Tumor development results from a variety of factors, including genetic factors, nutritional practices, lifestyle decisions, and environmental conditions [3, 4]. Although there have been improvements in treatment methods, the outlook for many cancer patients continues to be bleak owing to individual differences, tumor heterogeneity, and the inherent limitations of current therapies. Therefore, understanding the complex networks that drive tumor formation and cancer advancement, along with identifying biomarkers related to cancer progression, is crucial for improving patient survival rates and the chances of early recovery.

Recent developments in next-generation sequencing methodologies, along with the application of bioinformatics in oncology research, have greatly enhanced our understanding of how cancer starts and progresses, especially at the molecular and genetic levels. A growing number of non-coding RNAs (ncRNAs) have been recognized as crucial contributors to tumor development [5, 6]. Among the various types of ncRNAs, long non-coding RNAs (lncRNAs) have often been found to be dysregulated across a range of cancers, providing valuable insights for discovering biomarkers and potential therapeutic strategies. LncRNA is defined as a transcript that exceeds 200 nucleotides and is classified as an RNA molecule that does not code for proteins [7]. Initially, because of its non-coding nature, it was regarded as trivial transcriptional byproduct from non-functional genes and received minimal focus. However, recent research has shown that lncRNAs interact with numerous genes, proteins, DNA, or RNA entities, significantly influencing the onset and evolution of tumors by regulating various biological pathways, including proliferation, migration, invasion, cell cycle control, cell survival, drug resistance, and epithelial-mesenchymal transition (EMT) [8–10]. As a result, understanding the complex characteristics of lncRNAs, their impacts on various cancer-associated physiological mechanisms, and their interplay with other biomolecules is crucial for evaluating their potential regulatory functions in cancer and their prospective applications in personalized medicine.

Tumor protein 53 target gene 1 (TP53TG1), a recently identified lncRNA, was first discovered in a colon cancer cell line known as SW480-LOWTP53-1 [11]. Extensive studies have indicated that TP53TG1 may function as either an oncogene or a tumor suppressor in different tumors, influencing various cancer-related processes, including regulation of the cell cycle, proliferation, migration, invasion, EMT, as well as resistance to radiation and drugs. The abnormal expression of TP53TG1 is also closely related to tumor size, pathological stage and patient prognosis.

This article provides a review of the biological roles, clinical implications, and molecular mechanisms associated with TP53TG1, with the goal of enhancing our understanding of its diverse functions in oncology while highlighting its potential as a significant biomarker and therapeutic target.

## Characteristics of TP53TG1

**Fig. 1:**
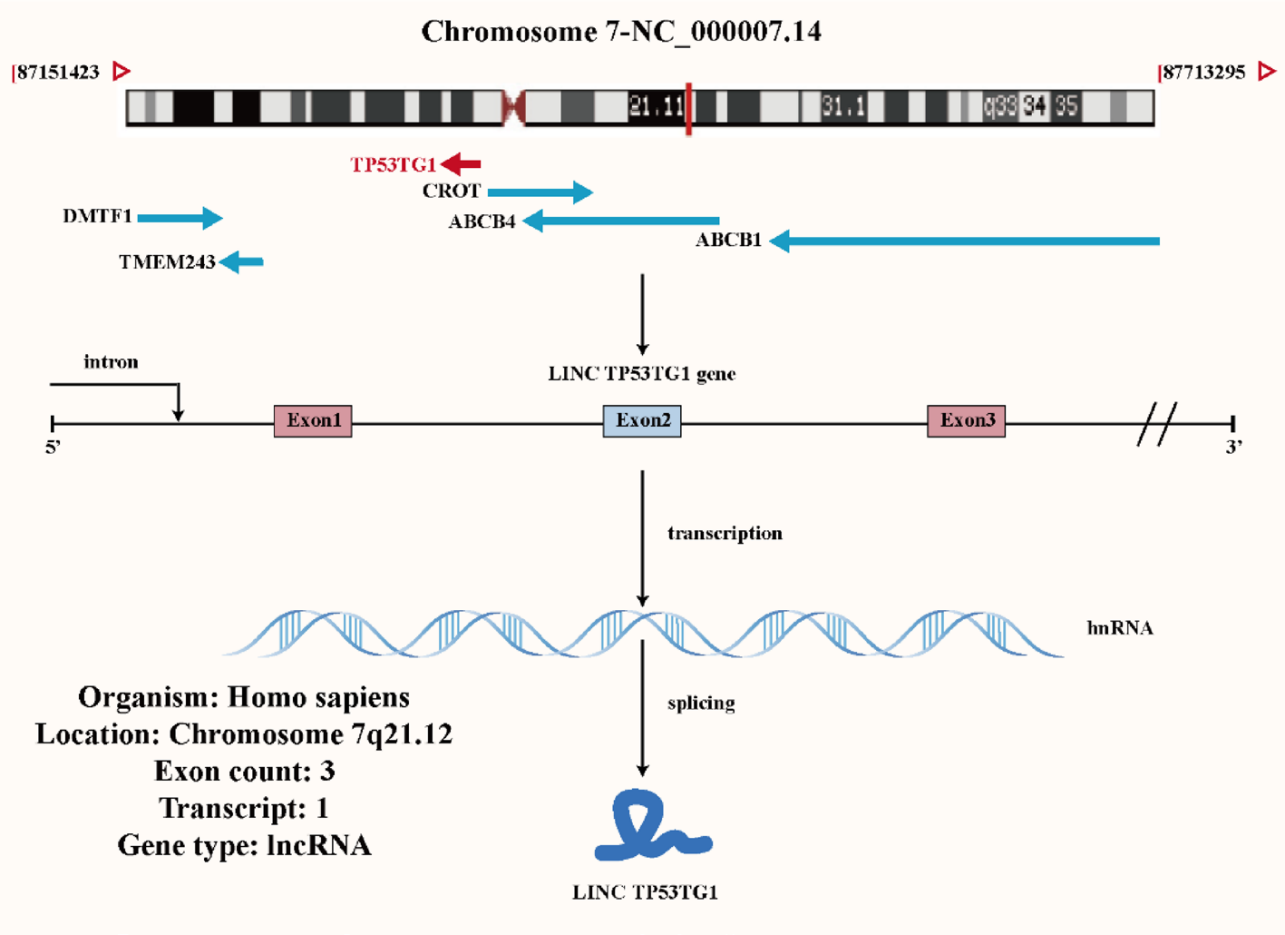
TP53TG1 is located on human chromosome chromosomal region 7q21.12 and comprises 3 exons, with the potential to generate up to 1 transcript variant according to the NCBI database (the chromosome map is provided by the Genecard website, while the rest is created using the Adobe illustrator 2024)

**Fig. 2:**
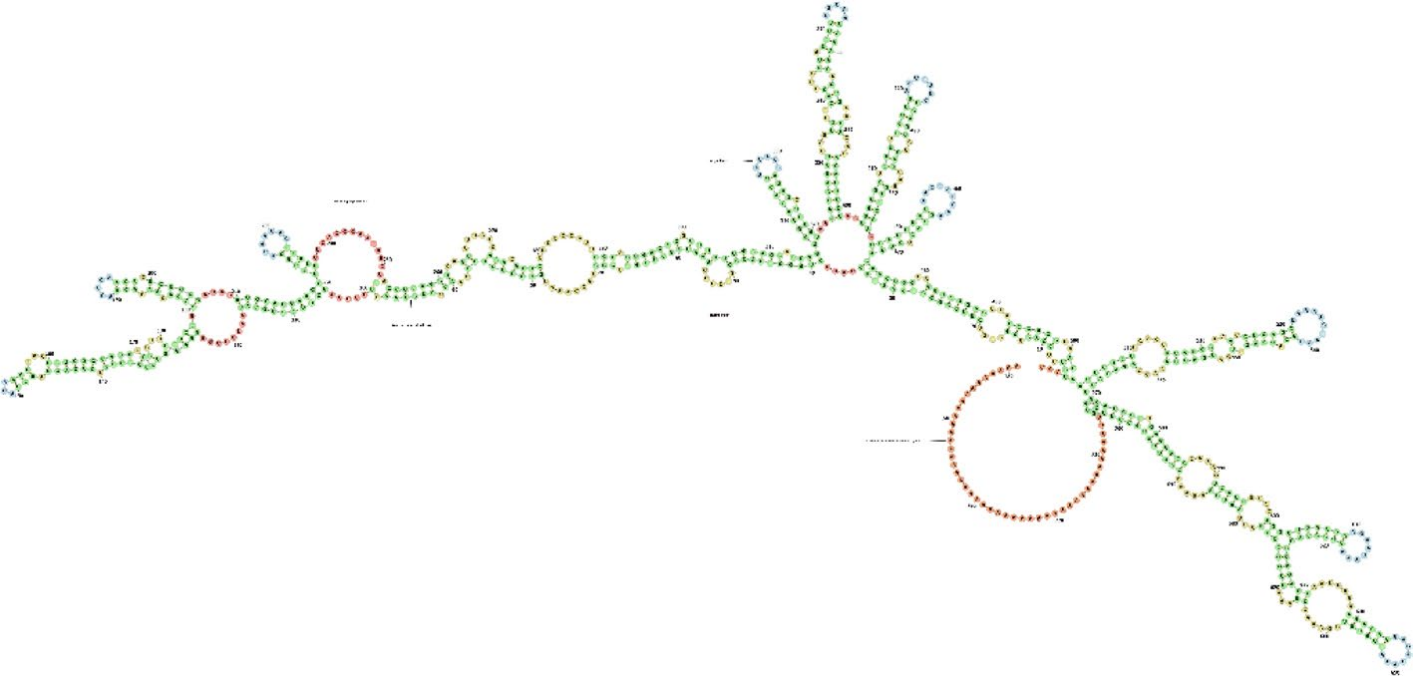
Predicted secondary structure of the TP53TG1 RNA (http://rna.tbi.univie.ac.at/forna/). The nucleotides are colored according to their structural context within the RNA fold: green represents stems (canonical helices); red indicates multiloops (junctions); yellow denotes internal loops; blue marks hairpin loops; and orange highlights the 5’ and 3’ unpaired terminal regions. The 5’ to 3’ polarity proceeds from left to right

**Fig. 3:**
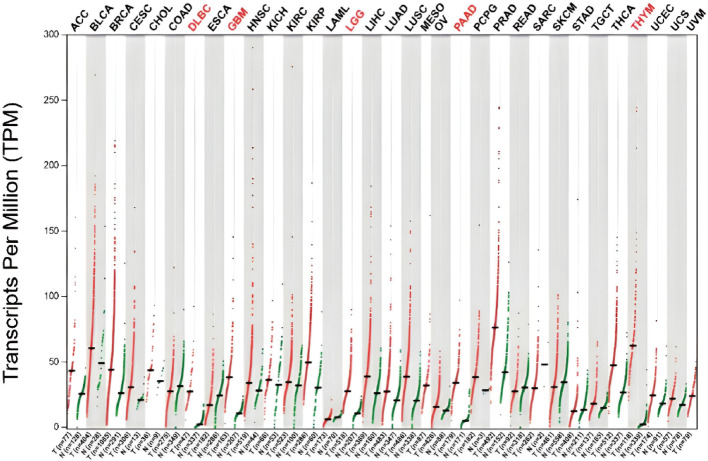
The expression of TP53TG1 in various tumors was different from that in normal tissues. Log2FC: 1; Q value: 0.01. (data from GEPIA (Gene Expression Profiling Interactive Analysis)). N, Normal tissue, T, tumor tissue. ACC, Adrenocortical carcinoma; BLCA, Bladder Urothelial Carcinoma; BRCA, Breast invasive carcinoma; CESC, Cervical squamous cell carcinoma and endocervical adenocarcinoma; CHOL, Cholangio carcinoma; COAD, Colon adenocarcinoma; DLBC, Lymphoid Neoplasm Diffuse Large B-cell Lymphoma; ESCA, Esophageal carcinoma; GBM, Glioblastoma multiforme; HNSC, Head and Neck squamous cell carcinoma; KICH, Kidney Chromophobe; KIRC, Kidney renal clear cell carcinoma; KIRP, Kidney renal papillary cell carcinoma; LAML, Acute Myeloid Leukemia; LGG, Brain Lower Grade Glioma; LIHC, Liver hepatocellular carcinoma; LUAD, Lung adenocarcinoma; LUSC, Lung squamous cell carcinoma; MESO, Mesothelioma; OV, Ovarian serous cystadenocarcinoma; PAAD, Pancreatic adenocarcinoma; PCPG, Pheochromocytoma and Paraganglioma; PRAD, Prostate adenocarcinoma; READ, Rectum adenocarcinoma; SARC, Sarcoma; SKCM, Skin Cutaneous Melanoma; STAD, Stomach adenocarcinoma; TGCT, Testicular Germ Cell Tumors; THCA, Thyroid carcinoma; THYM, Thymoma; UCEC, Uterine Corpus Endometrial Carcinoma; UCS, Uterine Carcinosarcoma; UVM, Uveal Melanoma

**Fig. 4:**
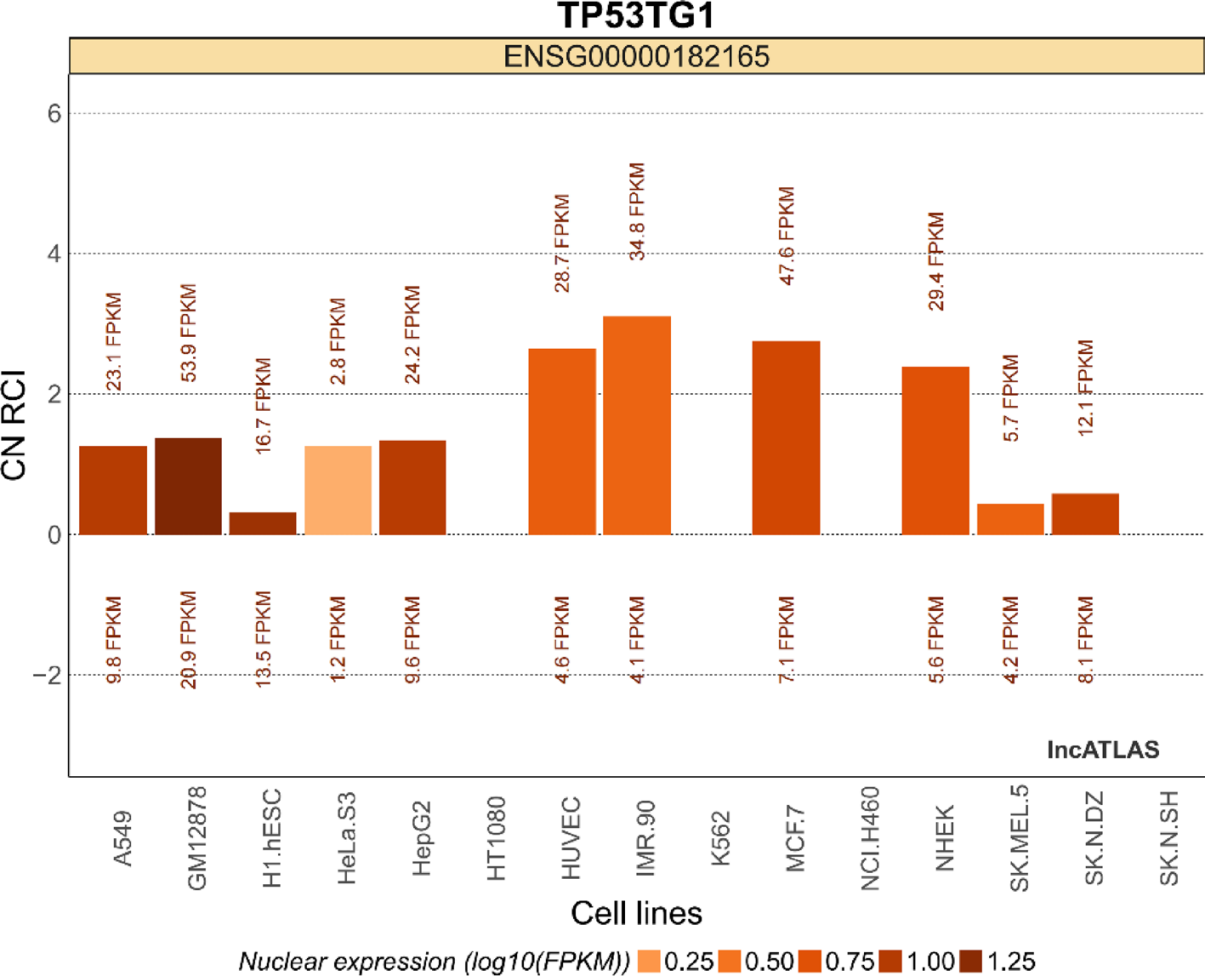
The localization of TP53TG1 in common cell lines is mainly located in the cytoplasm (bar plot from lncATLAS analysis; https://lncatlas.crg.eu/), and its subcellular localization is determined using RCI (Relative Concentration Index) and expression values for cytoplasmic/nuclear distribution

The TP53TG1 is located on chromosome region 7q21.12 of the human genome. It has three exons and generates one variant of transcript (Fig. 1, https://www.ncbi.nlm.nih.gov/gene/11257). To investigate the secondary structure of lncRNA TP53TG1 in depth, this study utilized the ViennaRNA Web Services (Fig. 2, http://rna.tbi.univie.ac.at/forna/) to visualize it through a force-directed graph layout method. An analysis using the GEPIA online database (http://gepia.cancer-pku.cn/) revealed that the expression of TP53TG1 varies significantly across several cancers, including diffuse large B-cell lymphoma (DLBC), glioblastoma multiforme (GBM), lower grade glioma (LGG), pancreatic adenocarcinoma (PAAD), and thymoma (THYM) (Fig. 3). Furthermore, the analysis of RCI and expression values across all cell types in the lncATLAS database (http://lncatlas.crg.eu/) indicates that TP53TG1 is primarily localized in the cytoplasm (Fig. 4).

## Role of TP53TG1 in various cancers

**Fig. 5:**
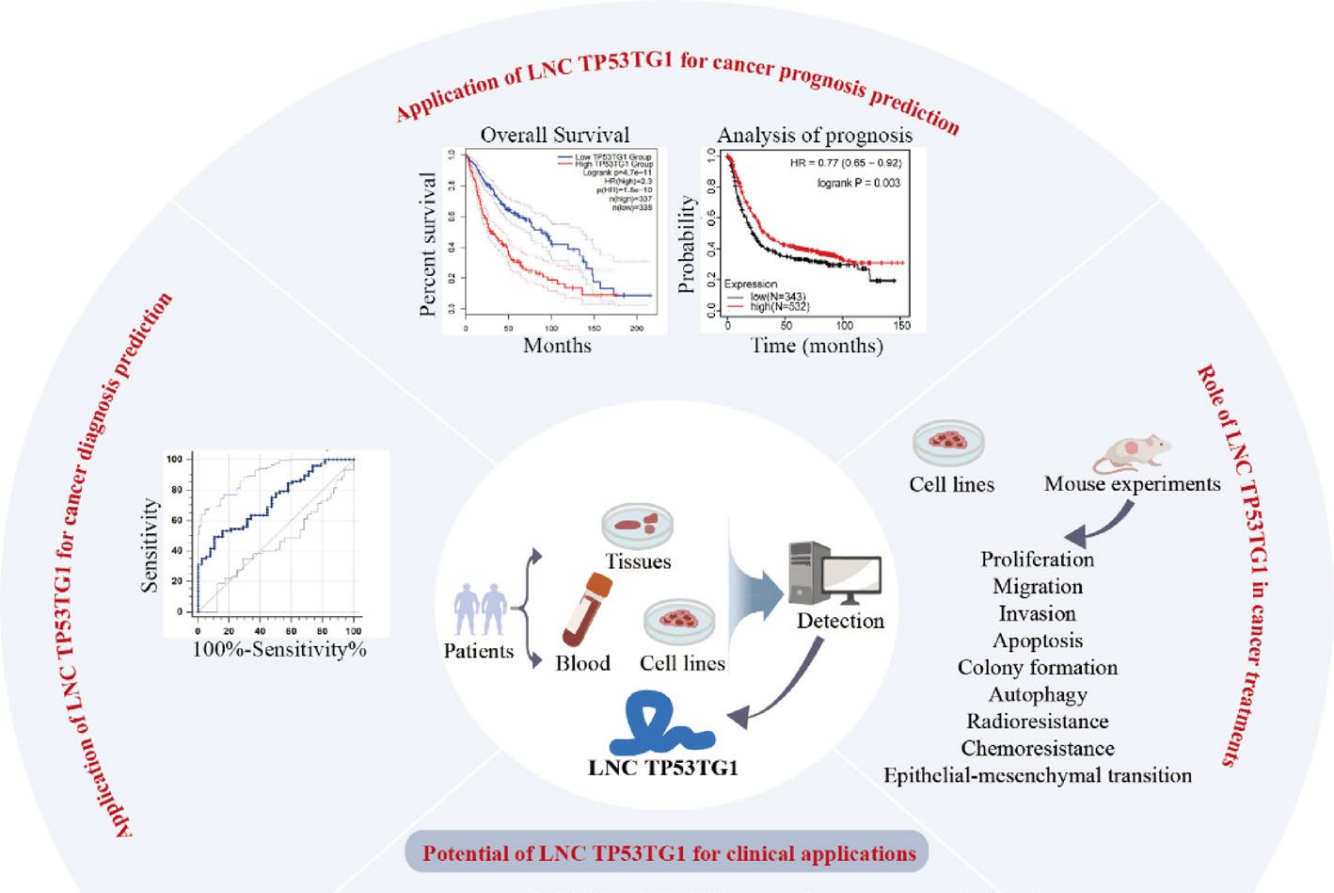
Overview of LncRNA TP53TG1 clinical applications and mechanisms in cancer. This schematic illustrates TP53TG1 as a biomarker and therapeutic target. (Center) Detectability in patient tissues, blood, and cell lines. (Left) Diagnostic accuracy demonstrated by ROC curve analysis. (Top) Prognostic correlations with tumor stage, metastasis, and recurrence; Kaplan-Meier survival analysis supports risk stratification. (Right) Functional impacts on malignant phenotypes-including proliferation, migration, invasion, apoptosis, autophagy, EMT, and chemoradioresistance-validated by in vitro and in vivo experiments

TP53TG1 exhibits abnormal expression patterns in various tumor tissues and has a strong correlation with the clinical and pathological characteristics of cancer patients (Fig. 5; Table 1). In addition, TP53TG1 is involved in the progression of cancer by participating in various necessary biological processes (Tables 2 and 3).

**Table 1:** Expression patterns and clinical significance of TP53TG1 across human malignancies

Cancer type	Numbers of clinical samples	Expression in Tumor vs. Normal	Clinical characteristics	Survival Analysis	Diagnostic Utility	Study Design Annotation	References
Hepatocellular carcinoma	80	Downregulation	Tumor size, multiple tumors, BCLC stage and overall survival	OS (*p* = 0.018)	-	Discovery only	[15]
Gastric cancer	82	Downregulation	Tumor diameter, differentiation, TNM stage, lymph node metastasis stage and overall survival rate	Kaplan-Meier analysis revealed that GC patients with lower TP53TG1 expression had a dismal prognosis (HR = 0.77, logrank *P* = 0.003)	-	Discovery only	[23]
Luminal subtypes of breast cancer	115	Upregulation	lymph-node invasion	-	ROC curve (*p* = 0.731)	Subtype-specific discovery	[27]
Breast cancer	20	Downregulation	-	-	ROC curve (*p* = 0.0011)	Small cohort; needs validation	[29]
Colorectal cancer	67	Upregulation	TNM stage	-	-	Discovery only	[37]
Non-small cell lung cancer	40	Downregulation	TNM stage	-	-	Discovery only	[41]
Glioma	20	Upregulation	-	OS (*p* <0.01)		discovery-only cohort	[50]
Glioma	51	Upregulation	Tumor size, TNM stage, Lymph node metastasis	-	-	Moderate cohort; no validation	[51]
Glioma	6	Upregulation	TNM stage	-	-	discovery-only cohort	[55]
Glioma	-	Upregulation	-	OS (*p* <0.01)	-	Discovery only	[56]
Retinoblastoma	70	Upregulation	overall survival	OS (*P* = 0.00382)	-	Single-center cohort	[61]

**Table 2:** Impact of TP53TG1 on the development and metastasis of cancer xenografts

Cancer type	Animal models	Function	References
Hepatocellular carcinoma	Four-week-old male athymic BALB/c nude mice	↑↑TP53TG1: ↓Tumor growth, ↓lung metastatic nodules	[15]
Gastric cancer	Nude mice	↑↑TP53TG1: ↓Tumor weight, ↓tumor volume and ↓ pulmonary metastasis	[23]
Colorectal cancer	4−6-week-old male BALB/c mice	↑↑TP53TG1: ↓Tumor weight and volume	[35]
Non-small cell lung cancer	4−5-week-old Male BALB/c-nude mice	↑↑TP53TG1: ↓Tumor growth	[41]
Cervical cancer	6-week-old BALB/c female nude mice	↓↓TP53TG1: ↓Tumor volume and size	[47]
Glioma	4−6-week-old Female nude mice	↑↑TP53TG1: ↓Tumor growth	[50]
Retinoblastoma	4-week-old BALB/c nude mice	↓↓TP53TG1: ↓Tumor volume and size	[61]

↑↑ TP53TG1: ↑↑ means TP53TG1 overexpression; ↑ : ↑ means promoting. ↓↓ TP53TG1: ↓↓ means TP53TG1 knockdown or knockout; ↓ : ↓ means inhibiting

**Table 3:** Roles and mechanisms of TP53TG1 in multiple tumors

Cancer type	Cell lines	Expression	Effect in vitro	Regulatory mechanism	Role	Reference
Hepatocellular carcinoma	HepG2, SK-hep1, Hep3B, HCCLM3, Huh7	Downregulation	invasion↓,migration↓ proliferation↓ and EMT↓	Wnt/β-catenin pathway	Tumor Suppressor	[15]
Hepatocellular carcinoma	HepG2, PLC/PRF/5	Upregulation	migration↑, proliferation↑	ERK pathway	Oncogene	[16]
Hepatocellular carcinoma	HepG2, PLC/PRF/5, Hep3B	Upregulation	migration↑, proliferation↑	ERK pathway	Oncogene	[18]
Gastric cancer	AGS, MKN28, BGC823, HCG27, MGC803, MKN45	Downregulation	invasion↓, proliferation↓ and EMT↓	PI3K/AKT pathway	Tumor Suppressor	[23]
Colorectal cancer	CL−0071	Downregulation	proliferation↓ and apoptosis↑	TP53TG1/STAT	Tumor Suppressor	[35]
Colorectal cancer	SW480	Upregulation	migration↑, invasion↑, proliferation↑, EMT↑ and apoptosis↓	TP53TG1/miR−330−3p	Oncogene	[36]
Colorectal cancer	SW620, NCM460	Upregulation	invasion↑, proliferation↑ and EMT↑	TP53TG1/miR−18a/CDC42	Oncogene	[37]
Non-small cell lung cancer	A549	Downregulation	cisplatin sensitivity↓ and apoptosis↑	TP53TG1/miR−18a/PTEN	Oncogene	[41]
Cervical cancer	Hela	Upregulation	apoptosis↑	-	Tumor Suppressor	[46]
Cervical cancer	SiHa, Caski, HeLa, C33A	Upregulation	migration↑, invasion↑, proliferation↑, and apoptosis↓	PI3K/AKT/mTOR pathway	Oncogene	[47]
Glioma	U251, LN229	Upregulation	proliferation↑ and EMT↑	TP53TG1/miR−96−5p/STK17B	Oncogene	[50]
Glioma	LN229, T98G	Upregulation	proliferation↑, colony formation↑, autophagy↑, radioresistance↑, and apoptosis↓	TP53TG1/miR−524−5p/RAB5A	Oncogene	[51]
Glioma	U251, U87, LN18, A172	Upregulation	migration↑, proliferation↑, and apoptosis↓	glucose metabolism related genes	Oncogene	[52]
Retinoblastoma	SO-RB50, WERI-Rb1, Y79, RBL−13	Upregulation	migration↑, invasion↑, proliferation↑	TP53TG1/miR−33 b/SHCBP 1	Oncogene	[61]
Nasopharyngeal carcinoma	PANC−1, MIA PaCa−2	Upregulation	migration↑, invasion↑, proliferation↑	TP53TG1/miR−96	Oncogene	[63]

↑ : ↑ means promoting; ↓ : ↓ means inhibiting

### Hepatocellular carcinoma

Hepatocellular carcinoma (HCC) ranks as the fifth most prevalent cancer globally and stands as the fourth leading cause of cancer-related mortality worldwide, exhibiting a 5-year survival rate as low as 18.1% [12]. The malignant transformation leading to HCC is a multifaceted process influenced by a range of genetic and external environmental factors, making early detection and intervention vital for enhancing treatment outcomes. While recent progress in surgical techniques and liver transplantation has led to improved short-term survival rates, the prognosis for HCC remains bleak due to recurrence post-surgery and potential metastasis [13, 14]. As a result, further investigation into the molecular mechanisms involved and the identification of novel therapeutic targets are essential for developing new and effective treatments for HCC.

Notably, TP53TG1 exhibits significant downregulation in both HCC tissue samples and hepatoma cell lines, with patients exhibiting high levels of TP53TG1 showing a better 5-year survival rate compared to those with lower expression levels. Additionally, research has indicated that TP53TG1 interacts directly with peroxiredoxin-4 (PRDX4), facilitating its ubiquitin-mediated degradation, which in turn leads to the inhibition of the Wnt/β-catenin signaling pathway in HCC cells, thereby exerting an anti-tumor effect [15]. Conversely, other investigations have suggested that overexpression of TP53TG1 may trigger the ERK signaling pathway, resulting in the enhanced proliferation and migration of HCC cells [16]. Although sorafenib, as a multi-target kinase inhibitor, is one of the most effective single-agent therapeutic drugs for HCC, most patients develop drug resistance after 4–5 months, and there are also hidden risks of poor efficacy and toxic side effects [17]. Treatment with sorafenib resulted in an upregulation of TP53TG1 expression in HCC cells, while the loss of TP53TG1 conferred sensitivity to the anti-proliferative effects of sorafenib. Thus, combining sorafenib with the knockout of TP53TG1 could represent the most effective therapeutic strategy for managing HCC [18]. In conclusion, TP53TG1 plays a complex role in HCC, which can function as a tumor suppressor gene by inhibiting the Wnt/β-catenin signaling pathway, or as a proto-oncogene by activating the ERK signaling pathway to promote tumor development. Its specific function depends on the cell type, etiology and molecular mechanism. Future research should focus on the functional regulatory mechanism of TP53TG1 and its application potential in HCC.

### Gastric cancer

Gastric cancer (GC) represents a primary malignant tumor arising from the stomach’s epithelial cells and is the fifth leading cause of cancer-related deaths globally [19]. The majority of individuals diagnosed with GC are at an advanced stage, primarily due to the lack of early warning signs and the infrequent occurrence of routine screenings, often showing signs of invasion or metastasis. Although there have been remarkable advancements in both diagnostic methods and treatment alternatives, the outlook for GC patients continues to be grim, especially for those with advanced or recurrent types that cannot be surgically removed, resulting in very low survival rates. While pathological assessment is considered the gold standard, it comes with drawbacks, including invasiveness and the limitations inherent in tissue sampling [20]. Additionally, conventional clinical markers such as carcinoembryonic antigen (CEA), carbohydrate antigen (CA) 199, and CA724 exhibit insufficient sensitivity and specificity for effective diagnosis [21]. Hence, there is a critical need to discover diagnostic markers that are both highly sensitive and specific for the early identification of GC. studies have indicated that the overexpression of TP53TG1 can substantially decrease both the weight and volume of tumors. Irregularities in enzymes or proteins involved in m6A methylation modifications can lead to various diseases. In the context of cancer, the m6A methyltransferase facilitates the methylation of oncogenes and tumor suppressor RNA, while a subsequent group of m6A reading proteins functions to recognize these modifications, either enhancing or inhibiting the expression of oncogenes or tumor suppressor genes, thereby influencing cancer progression [22]. Data from bioinformatics sources and MeRIP experiments have identified a significant enrichment of m6A modifications on TP53TG1 in GC cells. The m6A recognition protein AlkB Homolog 5 (ALKBH5) was found to reduce the m6A modification levels of TP53TG1, impacting RNA stability. This suggests that the reduction of TP53TG1 in GC may be associated with processes involving m6A modifications [23].

### Breast cancer

Breast cancer (BC) is one of the most commonly diagnosed cancers worldwide and stands as the second leading cause of cancer-related deaths among women. It is projected that there will be approximately 310,720 new instances of female BC in 2024 [24]. Timely diagnosis is crucial for enhancing BC prognosis, and the identification of early diagnostic markers holds significant importance for this purpose. Fibroblasts linked to BC are crucial in the initiation and development of BC, with cancer-associated fibroblasts (CAFs) being the primary cellular elements found in the BC microenvironment [25]. The creation of lncRNA (FibLnc) scores that are specific to CAFs can forecast differences in the overall survival time of individuals with BC. TP53TG1 plays a role in shaping the BC microenvironment and may indirectly encourage tumor growth and spread by affecting CAF functions. Studies suggest that higher levels of TP53TG1 are generally correlated with a worse prognosis [26]. As a critical lncRNA, TP53TG1 displays significantly elevated expression in Luminal A and Luminal B subtypes when compared to Her2 and Basal-like types, and its overexpression is particularly associated with BC samples that test positive for estrogen receptor (ER) and progesterone receptor (PR). In addition, the expression of TP53TG1 is closely linked to clinical characteristics, including survival rates and lymph node metastasis in BC patients [27]. The expression levels of TP53TG1 are affected by the status of DNA methylation; it tends to rise when DNA methylation is low and is suppressed under conditions of hypermethylation. In the context of BC, TP53TG1 activation is associated with wild-type TP53. Moreover, TP53TG1 hinders the transcription of phosphoinositide 3-kinase (PI3K) by interacting with the Y-box binding protein 2 (YBX2) protein, thus influencing the phosphoinositide 3-kinase/protein Kinase B (PI3K/AKT) signaling pathway [28]. In patients diagnosed with triple-negative breast cancer, a significant reduction in TP53TG1 expression was observed, particularly when compared to individuals with non-triple-negative breast cancer. Furthermore, it was noted that the levels of TP53TG1 expression in patients increased significantly following chemotherapy, in contrast to their pre-chemotherapy levels. This finding suggests that TP53TG1 functions as a tumor suppressor in BC. Its expression is correlated with both cancer type and the response to chemotherapy [29]. In summary, factors such as estrogen receptor/progesterone receptor (ER/PR) status, lymph node involvement, and molecular subtypes influence TP53TG1 expression in BC, with its roles primarily associated with the TP53 signaling pathway, chemosensitivity, and epigenetic mechanisms. Further investigation into the precise regulatory mechanisms of TP53TG1, along with its interactions within other molecular networks, is warranted. Additionally, the development of non-invasive detection techniques based on TP53TG1 could enhance the effectiveness of early diagnosis and treatment monitoring for breast cancer.

### Colorectal cancer

Colorectal cancer (CRC) ranks third globally in terms of incidence and mortality rates [30]. The widespread use of colonoscopy has led to satisfactory diagnostic rates; however, it remains an invasive procedure. Alarmingly, the incidence of CRC in individuals under 50 years old is rising, particularly concerning left-sided colon cancer [31]. Studies have shown that lncRNAs play a crucial role in the occurrence and development of CRC, such as lncRNA ITGB8-AS1 and lncRNA POU6F2-AS1 [32, 33]. Importantly, the role of TP53TG1 in CRC has also been confirmed. TP53TG1 was first discovered by Takei et al. from CRC cell lines in 1998. It plays a significant role in the TP53 signaling pathway, DNA repair, and feedback mechanisms of cell damage [11]. Sphingomyelin-based nanoemulsions (DSNs) exhibit excellent physical and chemical stability, along with a strong capacity to bind nucleic acids. The development of effective TP53TG1 delivery systems utilizing nanosystems (pc TP53TG1-DSNs) can diminish the proliferation and migration rates of the HTC-116 CRC cell line, as well as their ability to form colonies, thereby demonstrating anti-tumor properties [34]. Ji et al. found that overexpression of TP53TG1 in CT26 cells would further enhance the inactivation of the STAT pathway, thereby reducing the production of PD-L1, enhancing the lethality of T cells and NK cells, reducing the immunosuppression of Treg, and limiting the occurrence and development of CRC [35]. Conversely, a study revealed that TP53TG1 was highly expressed in CAFs obtained from CRC tissues and their derived exosomes. This elevated TP53TG1 expression enhanced CRC cell activity and EMT through the targeting of miR-330-3p, thereby facilitating the progression of CRC [36]. In summary, TP53TG1 exhibits a complex duality in its role concerning the progression of CRC, as it can either promote or inhibit tumor development. This duality can be attributed to a range of regulatory factors that influence its function. These factors include the stage of tumor development, the status of the surrounding microenvironment, and variations inherent in the experimental models employed. To improve the clinical applicability of TP53TG1 in CRC treatment, future investigations should emphasize the need for individualized analyses and delve into the dynamic regulatory mechanisms that govern its action.

### Non-small cell lung cancer

Lung cancer represents the most prevalent form of cancer and is the foremost cause of cancer-related deaths globally. In 2020, lung cancer accounted for approximately 1.7 million fatalities [37]. Non-small cell lung cancer (NSCLC), recognized as the principal histological variant, has witnessed considerable advancements in treatment over the last two decades; however, the overall 5-year survival rate remains disappointingly low, particularly for metastatic cases [38, 39]. This highlights the urgent need for further investigations aimed at enhancing the prognosis for NSCLC. Bioinformatics assessments have identified TP53TG1 as one of the lncRNAs that is differentially expressed in NSCLC, with its expression notably reduced in this type of cancer [40]. Through Reverse Transcription-quantitative Polymerase Chain Reaction (RT-qPCR) analysis, Xiao et al. corroborated that TP53TG1 expression was significantly decreased in NSCLC tissues and cell lines [41]. Currently, Diaminodichloroplatinum (cisplatin, DDP) is broadly utilized in treating numerous human malignancies, including NSCLC. Nevertheless, patients with NSCLC who initially respond well to DDP frequently develop resistance to the drug, resulting in treatment failure [42]. The overexpression of TP53TG1 has been shown to increase the sensitivity of the DDP-resistant A549/DDP cell line to DDP. Additional research has demonstrated that TP53TG1 enhances DDP sensitivity in NSCLC by competing with miR-18a for the regulation of PTEN expression, thereby revealing a novel strategy to boost the effectiveness of chemotherapy in NSCLC cases [41]. Among female NSCLC patients, particularly those with lung adenocarcinoma (LUAD) subtype, the levels of TP53TG1 increase with the number of smoking pack years; however, no significant correlation has been observed in male LUAD patients. This suggests that TP53TG1 is a critical molecule through which smoking influences the development of LUAD in female patients [43]. In summary, TP53TG1 plays a role in inhibiting the progression of NSCLC and holds potential for therapeutic applications, yet current research remains limited, necessitating further study to investigate its full implications.

### Cervical cancer

Cervical cancer (CC) is one of the most prevalent malignant tumors among women worldwide, with nearly all cases attributed to human papillomavirus (HPV) infection [44]. In recent years, many countries have implemented HPV vaccination programs and conducted CC screenings using major HPV tests, followed by the treatment of precancerous lesions. These measures have proven highly effective in preventing and controlling the progression of CC. However, the incidence and mortality rates of CC in developing countries remain alarmingly high, primarily due to issues related to chemotherapy resistance and metastasis [45]. Therefore, it is essential to explore more effective treatment strategies for CC comprehensively. Pan-cancer analysis using the TCGA database has revealed that TP53TG1 is significantly upregulated in CC, with high expression levels associated with improved overall survival rates. Additionally, overexpression of TP53TG1 in the HeLa CC cell line inhibited cell proliferation and increased apoptosis, suggesting a potential tumor suppressive effect [46]. Liao et al. also demonstrated that TP53TG1 is significantly overexpressed in CC tissues and cells. However, contrary to previous findings, overexpression of TP53TG1 was found to significantly enhance cell proliferation, migration, and inhibit apoptosis, indicating a promoting effect on CC [47]. The contradictory role of TP53TG1 in CC is essentially due to its manifestation as an environment-dependent molecule. In the future, TP53TG1 should be placed in a dynamic regulatory network and combined with molecular pathological typing to endow it with precise biological significance in CC.

### Glioma

Glioma is among the most prevalent forms of primary malignant tumors affecting the nervous system, representing roughly 40% to 60% of tumors within the central nervous system. Despite significant advancements in neuro-oncology, the aggressive growth and high invasiveness of glioma cells have resulted in a grim prognosis and a consistently elevated mortality rate for individuals diagnosed with glioma [48, 49]. It is crucial to delve into the molecular mechanisms underlying glioma development and to identify reliable targets for diagnosis and treatment, as these are pressing matters that require immediate attention. TP53TG1 is predominantly expressed in glioma tissues and has a strong association with unfavorable patient outcomes. Research findings indicate that the suppression of TP53TG1 expression notably reduces both the proliferation of glioma cells and the EMT process. More specifically, TP53TG1 influences the expression of the target protein serine/threonine kinase 17B (STK17B) through competitive binding to miR-96-5p, thereby affecting the advancement of glioma. When the levels of TP53TG1 are decreased, miR-96-5p is able to more efficiently target and lower the expression of STK17B, which in turn hinders the proliferation and EMT of glioma cells [50]. Moreover, elevated levels of TP53TG1 are significantly associated with larger tumor sizes, advanced TNM staging, and lymph node metastasis. Validation through dual-luciferase reporter assays and RNA immunoprecipitation assays demonstrated that TP53TG1 functions as a “sponge” for miR-524-5p, enhancing the expression of member RAS oncogene family (RAB5A). This mechanism contributes to increased cell proliferation and autophagy while simultaneously reducing apoptosis and bolstering resistance to chemotherapy [51].

Autophagy is a self-degradation process of cells that can help cancer cells survive in stressful environments and autophagy drugs can cause the death of glioma cells. Through microarray analysis of the glioma genome in China, it was found that TP53TG1 is an autophagy-related lncRNA, and the survival time of patients with high expression of it is significantly shorter than that of patients with low expression [52]. The endoplasmic reticulum to nucleus signaling 1 (ERN1) pathway of endoplasmic reticulum stress response signaling is related to the processes of apoptosis and cell death. Inhibiting the function of the ERN1 gene can significantly reduce tumor growth. L-glutamine deprivation can induce upregulation of TP53TG1 expression in U87 glioma cells. However, in ERN1 knockdown cells, this response is completely eliminated, indicating that the sensitivity of TP53TG1 to L-glutamine deprivation depends on the ERN1 signaling pathway [53]. Cancer cells prefer to convert glucose into lactic acid under aerobic conditions to support the biosynthetic precursor required for rapid growth. In solid tumors, due to insufficient blood supply, cancer cells are often in an environment of glucose deficiency, hypoxia and acidity [54]. Low-glucose treatment significantly upregulated the expression of TP53TG1, promoted the expression of glucose-regulated protein 78 (GRP78), lactate dehydrogenase A (LDHA), and isocitrate dehydrogenase 1 (IDH1) genes, inhibited the expression of pyruvate kinase M1 (PKM1) and pyruvate kinase M2 (PKM2), and enhanced the survival and migration ability of glioma cells in a low-glucose environment, suggesting that TP53TG1 may become a new target for the diagnosis or treatment of glioma by regulating glucose metabolism [55]. Remarkably, Gandhi et al. discovered that the levels of TP53TG1 expression are notably reduced in glioma cells and tissues [56]. Consequently, the varying levels of TP53TG1 in glioma indicate the tumor’s heterogeneity and the intricacy of its molecular mechanisms. Upcoming studies should aim to integrate multi-omics data, along with dynamic monitoring and functional validation, to elucidate its function across different pathological stages and establish a foundation for precision treatment.

### Other cancers

TP53TG1 is not only abnormally expressed in the aforementioned tumors but is also differentially expressed in various other malignancies, such as ovarian cancer (OC), esophageal cancer (EC), and pancreatic ductal adenocarcinoma (PDAC). Peritoneal metastasis is a prevalent form of metastasis in OC. In samples from OC patients with peritoneal metastasis, the methylation level of the TP53TG1 gene is significantly elevated, suggesting its potential role as a biomarker for OC progression and metastasis [57]. Insulin-like growth factor 2 (IGF2) is known to promote cell proliferation and migration, exhibiting high expression levels across numerous cancers, including EC [58]. TP53TG1 functions as a ‘molecular sponge’ by sequestering miR-6835, which leads to the upregulation of IGF2 expression and facilitates the malignant transformation of EC cells [59]. Six lncRNAs with the highest expression levels were identified from RNA-seq and ChIP-seq databases of nasopharyngeal carcinoma (NPC) cell lines, including LINC00152, NEAT1, MIR205HG, LINC00941, MALAT1, and TP53TG1. The knockdown of TP53TG1 using siRNA can inhibit the proliferation and clonogenic ability of NPC cells, indicating that TP53TG1 has a pro-cancer effect in NPC [60]. Furthermore, the overall survival of retinoblastoma (RB) patients with elevated TP53TG1 levels is shorter, with TP53TG1 expression significantly increased in RB tissues and cells. Further mechanistic studies have demonstrated that TP53TG1 enhances the proliferation, migration, and invasion abilities of RB cells by inhibiting miR-33b and upregulating SHC binding and spindle associated 1 (SHCBP1) expression [61]. Public gene expression profiling analyses (GSE16515 and GSE15471) revealed that TP53TG1 expression levels in PDAC tissues are significantly higher than those in normal pancreatic tissues. Results from RT-qPCR analysis of 15 pairs of PDAC and adjacent pancreatic tissue samples indicated a significant upregulation of TP53TG1 in PDAC tissues. Furthermore, it has been discovered that TP53TG1 functions as a ceRNA that upregulates KRAS expression by competitively binding to miR-96. This mechanism subsequently promotes the proliferation and invasion of PDAC cells [62, 63].

## Regulatory mechanism of TP53TG1 in human cancer

TP53TG1 influences cancer through multiple mechanisms, including the lncRNA-miRNA-mRNA ceRNA network and the activation of the STAT, PI3K/AKT, and Wnt/β-catenin signaling pathways. These interactions underscore the complex role of TP53TG1 in tumor biology.

**Fig. 6:**
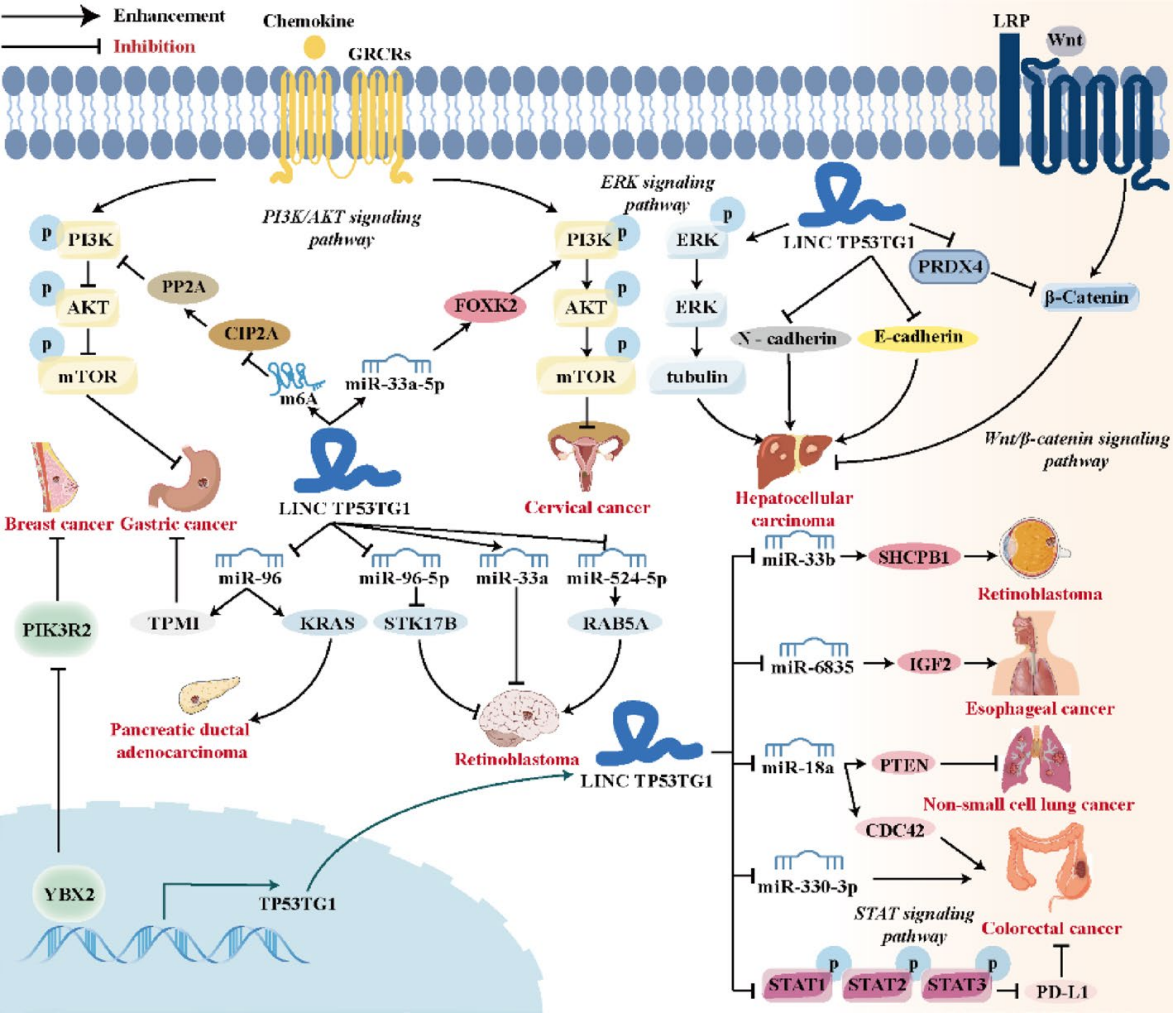
The molecular mechanisms of lncRNA TP53TG1 in different human cancers. The TP53TG1-centered ceRNA network comprises 9 miRNAs and modulate critical signaling pathways (e.g., PI3K/AKT, Wnt/β-catenin, STAT, etc.), thereby promoting or inhibiting cancer advancement

The ceRNA hypothesis has emerged as a focal point in RNA biology research in recent years, unveiling novel mechanisms of RNA interactions [64, 65]. LncRNAs reshape the miRNA-mRNA regulatory network via the ceRNA mechanism, serving as crucial hubs for gene expression regulation [66]. As shown in Fig. 6, TP53TG1 modulate the expression of its downstream target genes through competitive interactions with miRNAs, thereby exerting either carcinogenic or tumor suppressor effects in various human malignancies.

It has been reported that miR-330-3p [36] and miR-33a [56] are involved in the carcinogenic or anti-cancer activity of TP53TG1, but their downstream targets and their signaling pathways or biomolecular interactions remain to be further studied. Xiao et al. [41] confirmed the direct binding of TP53TG1 to miR-18a through dual-luciferase reporter gene assay, RNA pull-down assay and RNA immunoprecipitation assay. By inhibiting miR-18a, TP53TG1 indirectly promotes the expression of its target gene PTEN, thereby enhancing the sensitivity of NSCLC cells to cisplatin. In glioma, TP53TG1 regulates the target protein STK17B by competitively binding to miR-96-5p, thus regulating the progression of glioma [50]. RAB5A is a member of the Rab GT3 family and participates in the regulation of cell biological functions, having become an important regulatory factor for various cancers [67]. TP53TG1 can upregulate the expression of RAB5A by adsorbing miR-524-5p, promoting radioresistance in glioma cells [51]. TP53TG1 adsorbs miR-33b through the 3’UTR region. miR-33b isolated by TP53TG1 can no longer act on the 3’UTR of its target gene SHCBP1, resulting in an increase in the mRNA and protein expression levels of SHCBP1, thereby promoting the progression of RB [61]. In addition, Zhang et al. found that TP53TG1 promotes the growth and progression of PDAC by competitionally binding to miR-96 as a ceRNA and regulating KRAS expression [63].

Immunotherapy targeting PD-1/PD-L1 is a new advancement in the field of tumor treatment in recent years. PD-L1 can inhibit the proliferation and activation of T cells by binding to its receptor programmed cell death 1 (PD-1), and negatively regulate the immune response process of the body, thereby mediating the immune escape of tumors and promoting tumor growth [68, 69]. In CRC, overexpression of TP53TG1 can significantly reduce the phosphorylation levels of STAT1, STAT2, and STAT3. Further experiments revealed that TP53TG1, as a tumor suppressor lncRNA, reduces PD-L1 expression by inhibiting the STAT signaling pathway, thereby relieving the immunosuppressive state in the tumor microenvironment, restoring the anti-tumor activity of T cells and NK cells, and ultimately inhibiting the progression of CRC [35]. The PI3K/AKT signaling pathway is one of the core pathways regulating tumorigenesis and development and its abnormal activation is closely related to the proliferation, apoptosis resistance, metabolic reprogramming and therapeutic resistance of various tumors [70, 71]. Shao et al. confirmed that TP53TG1 exerts an inhibitory effect on breast cancer by binding to YBX2 and blocking its transcriptional activation of phosphoinositide-3-Kinase regulatory subunit 2 (PIK3R2), a member of the PI3K family [28]. In GC, TP53TG1 binds to cellular inhibitor of protein phosphatase 2 A (CIP2A) and promotes its ubiquitination, thereby inhibiting the activation of the PI3K/AKT pathway and suppressing the development of GC [23]. PRDX4, as an antioxidant protein, is known to stabilize the β-catenin protein and promote its nuclear translocation, thereby activating the Wnt/β-catenin signaling pathway [72]. TP53TG1 inhibits the Wnt/β-catenin signaling pathway by reducing the level of PRDX4 protein and weakening the stability of β-catenin, thereby suppressing the proliferation, migration and EMT of HCC cells [15].

## Potential clinical applications of TP53TG1

Through extensive research on cancer, significant advancements in treatment approaches have markedly enhanced the survival rates of individuals affected by the disease. Nonetheless, the absence of suitable early detection methods for tumor biomarkers has resulted in persistently high mortality rates for many cancer types. Consequently, timely diagnosis and the implementation of effective treatment strategies can yield substantial advantages for cancer patients. LncRNAs are abundantly found in tumor tissues, plasma, and urine. Their regulatory functions in cancer offer valuable insights for accurate diagnosis and treatment, making them promising candidates for new biomarkers in cancer diagnosis, treatment, and prognosis [73]. For example, the lncRNA prostate cancer antigen 3 (PCA3) has received approval from the Food and Drug Administration as a dependable diagnostic biomarker for prostate cancer [74]. Likewise, TP53TG1 has emerged as a significant biomarker, showing great promise as both a biomarker and a therapeutic target for future diagnosis and prognosis across various cancer types.

### TP53TG1 as a potential diagnostic biomarker in cancers

Numerous investigations have revealed that TP53TG1 is significantly upregulated in various tumors, including CC [47], OC [57], EC [58], and PDAC [63]. Conversely, some studies indicate that the expression of TP53TG1 is comparatively lower in cancers such as NSCLC [41] and HCC [15]. The irregular expression of TP53TG1 in certain cancerous tissues has shown promise in differentiating malignant tissues from surrounding normal tissues, as observed in cases such as CC [47], glioma [55] and PDAC [63], which suggests the potential of TP53TG1 as a valuable marker for the early diagnosis of cancer. The logistic regression model demonstrated strong diagnostic capabilities. When analyzing 92 cancerous lung tissues and non-cancerous lung tissues diagnosed as NSCLC, it was found that TP53TG1 could accurately distinguish between cancerous and non-cancerous lung tissues (with an AUC value of 0.98 ± 0.01) [40]. The high stability of lncRNAs within circulation, along with their resistance to enzymatic breakdown, makes them more reliable biomarkers compared to other nucleic acids. Nevertheless, current research predominantly focuses on identifying the abnormal expression of TP53TG1 within tissue samples. Future investigations should aim to assess the levels of TP53TG1 in bodily fluids, including serum, plasma, and peripheral blood cells, for diagnosing malignant tumors. Additionally, integrating TP53TG1 with established cancer biomarkers could potentially enhance the precision and specificity of cancer diagnostics.

### TP53TG1 as a therapeutic target in cancers

Targeted therapies have the potential to effectively eliminate tumors and greatly enhance patient outcomes across various clinical settings by concentrating on specific and efficient targets [75]. Additionally, there is a growing interest in the exploration of lncRNA as a promising therapeutic target. Techniques for effective gene knockout or overexpression could generate innovative insights for targeted research on lncRNAs. According to the report, silencing TP53TG1 notably suppresses tumor growth in RB nude mice [61]. Knocking down TP53TG1 enhances the sensitivity of HCC cells to sorafenib, and combining the inhibition of TP53TG1 with a lower dose of sorafenib can increase the HCC therapeutic effect and reduce potential toxicity [18]. Another study by Fang et al. revealed that TP53TG1 inhibits the PI3K/AKT signaling pathway and slows down the progression of GC by interacting with CIP2A, promoting its ubiquitination and degradation [23]. Additionally, TP53TG1 heightened the sensitivity of NSCLC cells to cisplatin by modulating the miR-18a/PTEN axis, outlining a novel strategy to improve the effects of chemotherapy in NSCLC [41]. The therapeutic significance of TP53TG1 has also been established through its silencing or overexpression in various other malignant tumors, including HCC [16], CRC [35], and PDAC [63].

### TP53TG1 as a prognostic marker in cancers

The area under the curve (AUC) is regarded as the benchmark for assessing the discriminatory power of predictive models in tumor prognosis. A higher AUC value indicates a more dependable model for classifying patient risk. TP53TG1 is highly expressed in BC tissues, and AUC (0.731) analysis shows that it has moderate value for the prognosis of BC [27]. Likewise, the analysis using the cox proportional hazards regression model revealed that TP53TG1 exhibited increased levels in the BC group with the most favorable prognosis, whereas its expression was decreased in the BC group with a less favorable prognosis. This suggests that elevated TP53TG1 expression might be linked to better outcomes for patients with BC [28]. Additionally, an analysis of the Chinese Glioma Genome Atlas dataset revealed that patients with high TP53TG1 expression had significantly longer survival times compared to those with low expression. This finding underscores the ability of TP53TG1 expression levels to effectively differentiate patient survival risks, establishing it as a credible prognostic biomarker for glioma [52]. Similarly, low TP53TG1 expression is associated with more advanced clinical features such as tumor diameter, differentiation, TNM classification, and lymph node metastasis stage. Moreover, decreased TP53TG1 expression in GC patients is significantly associated with poor prognosis. These results indicate that TP53TG1 can be used as a potential indicator for the prognosis of GC [23]. Pan-cancer analysis shows that the higher the TP53TG1 expression, the higher the overall survival rate. In CC tissues, the expression of TP53TG1 is higher than that in normal tissues adjacent to the cancer, and the prognosis analysis indicates that high expression of TP53TG1 is associated with a favorable prognosis [46]. TP53TG1 has been identified as one of the important lncRNAs related to oxidative stress, the multivariate Cox analysis revealed that its high expression was significantly associated with a poor prognosis in patients with GC (*P* < 0.05) [76].

## Conclusion and future perspectives

In recent years, the newly discovered lncRNA TP53TG1 has demonstrated complex and multifaceted biological functions across various cancers, encompassing tumor initiation, progression, metastasis, drug resistance, EMT and immune regulation. Its core functional characteristic lies in its ability to act as both a tumor suppressor and an oncogene. TP53TG1 can promote the ubiquitination and degradation of PRDX4 in HCC cells through direct interaction, thereby inhibiting the Wnt/β-catenin pathway and exerting tumor-suppressive effects. Conversely, studies have shown that TP53TG1 can also enhance HCC cell proliferation and migration by activating the ERK pathway. This dual role underscores the high regulatory flexibility of such lncRNAs during tumorigenesis. Similar “double-edged sword” phenomena are particularly evident in CRC and glioma. In CRC, TP53TG1 can reside in CAFs and their exosomes, promoting tumor progression by sequestering miR-330-3p to upregulate CDC42 protein expression. Conversely, when TP53TG1 is overexpressed within tumor cells, it suppresses CRC immune evasion and progression by inhibiting the STAT signaling pathway and reducing PD-L1 expression. In glioma, TP53TG1 overexpression specifically sequesters miR-96-5p and miR-524-5p, thereby promoting the expression of the oncogenes STK17B and RAB5A. However, glioma cells with low TP53TG1 expression exhibit tumor-suppressive effects by targeting and negatively regulating miR-33a expression. These seemingly contradictory results stem not only from the heterogeneity of tumor cell lineages but also from the complexity of lncRNA’s functional regulatory mechanisms, dynamic gene expression patterns, and the selectivity of promoter methylation and silencing within cis-regulatory activities. All of the above reveal that the regulation of lncRNA is not a simple linear process but is instead achieved through a multi-layered molecular information platform.

Abnormal TP53TG1 expression also shows significant correlations with clinical pathological features such as TNM staging, lymph node metastasis, and Lauren classification in GC, making it a highly promising biomarker. HCC patients with high TP53TG1 expression demonstrate significantly better 5-year survival rates than those with low expression, indicating its potential as a prognostic indicator. TP53TG1 expression levels are also closely associated with tumor staging and chemotherapy response in GC, highlighting its importance as a predictor of chemosensitivity. However, in the Luminal subtype of breast cancer BC, high TP53TG1 expression may correlate with poor prognosis. These cancer-type-specific differences further emphasize the necessity of interpreting TP53TG1 as a biomarker in the context of specific cancer types and molecular classifications. Additionally, TP53TG1 expression changes in CRC and NSCLC are closely linked to PD-L1 regulation in the immune microenvironment and chemotherapy resistance to 5-FU and cisplatin, reinforcing its utility as a therapeutic response monitoring marker. Particularly in NSCLC, the association between TP53TG1 and female smoking history provides new insights for gender-specific lung cancer early warning.

Despite the remarkable progress made in the research on the lncRNA TP53TG1, numerous challenges and limitations persist. Firstly, although the functional heterogeneity of TP53TG1 as a biomarker has been extensively studied in both in vitro and in vivo experiments across different tissues and cells, standardized protocols for dynamic monitoring in human tumor tissues and cells have yet to be established. Secondly, most existing studies are confined to specific cancer types or single signaling pathways, lacking cross-cancer comparative analyses to comprehensively map its global regulatory network. Thirdly, the transcriptional interference processes and chromatin structures of TP53TG1 in tumors, as well as structural domains such as epigenetic complexes and spatial genome organization, remain to be further elucidated. Fourthly, the majority of studies have been limited by small sample sizes and retrospective analyses, lacking multi-center, large-sample prospective clinical cohort studies that are essential for rigorously validating the sensitivity, specificity, and predictive value of TP53TG1 as a diagnostic or prognostic biomarker.

In conclusion, to more comprehensively elucidate the biological functions and clinical potential of TP53TG1 in cancer, multi-cancer-type and multi-omics integrated analyses should be conducted to systematically compare its expression patterns, functional mechanisms, and clinical prognostic associations across different cancer types. By integrating genomic, transcriptomic, epigenomic, and proteomic data, a global regulatory network of TP53TG1 can be constructed, and multi-center, large-sample prospective clinical cohorts established to evaluate its application in early cancer diagnosis, therapeutic response prediction, and recurrence monitoring. Simultaneously, exploring combination diagnostic strategies with other biomarkers could enhance diagnostic accuracy such as CEA, CA199, PD-L1. Furthermore, to advance its application as a liquid biopsy marker, convenient detection methods for TP53TG1 expression in blood, urine, or exosome-based liquid samples should be developed, with sensitivity and specificity improved through novel detection platforms such as nano-integration technology and microfluidic chips. Therefore, future efforts require strengthened multidisciplinary collaboration to develop targeted therapeutic strategies regulating TP53TG1 expression, including small-molecule drugs, siRNA, and antisense oligonucleotides, based on its biological mechanisms. Promoting the deep integration of TP53TG1 basic research with clinical applications while exploring its synergistic effects in immunotherapy, chemotherapy sensitization, and gene therapy will advance its precision medicine applications, offering new hope for cancer patients.

## Data Availability

No datasets were generated or analysed during the current study.
